# Note-taking and science inquiry in an open-ended learning environment

**DOI:** 10.1016/j.cedpsych.2018.08.004

**Published:** 2018-10

**Authors:** Yang Jiang, Jody Clarke-Midura, Bryan Keller, Ryan S. Baker, Luc Paquette, Jaclyn Ocumpaugh

**Affiliations:** a Teachers College, Columbia University, 525 West 120th Street, New York, NY 10027, United States; b Utah State University, 2830 Old Main Hill, Logan, UT 84322, United States; c University of Pennsylvania, Graduate School of Education, 3700 Walnut Street, Philadelphia, PA 19104, United States; d University of Illinois at Urbana–Champaign, 1310 S. 6th Street, Champaign, IL 61820, United States

**Keywords:** Note-taking, Science inquiry, Multilevel analysis, Note-taking/reaccessing quantity and note content, Open-ended learning environments, Virtual Performance Assessments

## Abstract

Note-taking is important for academic success and has been thoroughly studied in traditional classroom contexts. Recent advancements of technology have led to more students taking notes on computers, and in different situations than are common in traditional instructional contexts. However, research on computer-based note-taking is still an emerging area, and findings from these studies are mixed. In this exploratory study, we conducted multilevel analysis to comprehensively investigate the relationship between note-taking measures and subsequent student success at science inquiry among middle school students, using two scenarios of an open-ended learning environment named Virtual Performance Assessments. Analysis revealed an advantage for content elaborative note-taking over content reproductive note-taking conditional on the source of notes taken, but other measures were less consistent between the two scenarios. Implications of the findings and limitations of this research are also discussed.

## Highlights

• Usage of digital notepad is related to performance in science inquiry tasks in OELE.
• Both taking and reaccessing notes facilitate science inquiry performance.
• Elaborative and reproductive notes’ relationship with success is content dependent.

## 1 Introduction

Note-taking, a common classroom practice and learning strategy, is both encouraged by educators and embraced by learners in various academic settings (Bonner and Holliday, 2006, Dunkel and Davy, 1989, Van Meter et al., 1994). According to a national survey of 5728 science and mathematics teachers across the United States, students took notes of lectures at least once a week in 54% of middle school science classes and 86% of high school science classes (Weiss, Banilower, McMahon, & Smith, 2001). Research has shown that taking and reviewing notes from lectures or texts are associated with positive learning outcomes (Armbruster, 2009, Kiewra, 1989, Peverly et al., 2007, Rickards and Friedman, 1978), with studies showing the importance of both the quantity and the content of notes (Bretzing and Kulhavy, 1979, Cohn et al., 1995, Fisher and Harris, 1973, Slotte and Lonka, 1999).

Many of these studies predate the rapid advancement of educational technology and the increasing use of computers in science education (Higgins & Spitulnik, 2008) and instead focus on the analysis of paper-based note-taking (e.g., Weiss et al., 2001). As more and more learning is happening through computer-based learning environments, researchers have begun to investigate the effects that computers have on students’ note-taking strategies and learning (e.g., Bauer and Koedinger, 2006, Bui et al., 2013, Crooks et al., 2007, Igo et al., 2005, McQuiggan et al., 2008, Mueller and Oppenheimer, 2014, Robinson et al., 2006). However, no study we are aware of has systematically and comprehensively investigated note-taking in science open-ended learning environments (OELEs) for middle school learners. This study explores the correspondence between note-taking within an OELE and success in scientific inquiry among middle school students.

### 1.1 Research on paper-based note-taking

As a popular, nearly ubiquitous academic strategy, note-taking has been thoroughly studied. In particular, there has been extensive research on traditional paper-based note-taking in the context of classroom lectures or learning from texts. Educational studies have long documented the crucial role of note-taking in facilitating academic success, especially for college students (Armbruster, 2009, Kiewra, 1989, Peverly et al., 2007). Researchers have identified two basic functions of note-taking that could explain its beneficial role in enhancing learning and performance – the encoding function (the process of recording information in notes supports the conversion of information to memory) and the external storage function (the use of notes as external memory storage that can be reviewed afterwards) (Di Vesta and Gray, 1972, Williams and Eggert, 2002).

#### 1.1.1 Encoding function

Several researchers have argued that the process of selecting and recording information in notes is, by itself, beneficial for learning and performance. They propose that taking notes promotes learning as it attracts learner attention to instructional content (Di Vesta and Gray, 1972, Einstein et al., 1985), facilitates translation of instructional content into text and one’s own understanding (Piolat, Olive, & Kellogg, 2005), enables better construction of deep-level mental representations of content (Bui et al., 2013, Slotte and Lonka, 1999), and empowers elaborative and generative processing by encouraging learners to connect new content with existing prior knowledge (Einstein et al., 1985, Peper and Mayer, 1978).

Note-taking is cognitively demanding and requires high cognitive effort as students have to process information, make decisions on which information to record, hold information temporarily in working memory while recording it and even organizing, paraphrasing, and elaborating on it (Bui et al., 2013, Piolat et al., 2005). Cognitive load is the amount of mental effort and requirements imposed on students’ limited working memory capacity (Sweller, 1994). Learning is limited if learners have to use resources in working memory for tasks that are not related to schema acquisition (e.g., extraneous cognitive load caused by instructional design of learning environments). The learning contexts where students study and take notes (e.g., lectures or text reading) typically impose high cognitive load (Kobayashi, 2005, Peverly et al., 2007, Piolat et al., 2005). As the information that originally needs to be stored in working memory has been stored in external storage (e.g., notebooks) during note-taking, the process of taking notes also offloads extraneous cognitive load imposed on students during learning (Moos, 2009, Piolat et al., 2005).

However, results of empirical studies on the benefits of encoding have been mixed (see Kiewra, 1985a, Kobayashi, 2005 for reviews). On the one hand, considerable research has indicated that students who took lecture notes (Barnett et al., 1981, Bretzing et al., 1987, Einstein et al., 1985) or text notes (Bretzing and Kulhavy, 1981, Lahtinen et al., 1997, Peverly et al., 2003, Rickards and Friedman, 1978) generally outperformed non-note-takers who merely listened to lectures or read texts on various tasks (e.g., comprehension, recall, retention) in the absence of reviewing notes, supporting the encoding function hypothesis with overall small to modest positive effects (Kobayashi, 2005). On the other hand, a number of other studies have shown no significant difference in performance between note-takers who did not review notes and non-note-takers (e.g., Howe, 1970, Kiewra et al., 1991), or have indicated that taking notes can even interfere with learning (e.g., Peck & Hannafin, 1983).

#### 1.1.2 External storage function

Findings of empirical studies testing the external storage function show higher consensus in favor of this hypothesis than research on the encoding function (Kiewra, 1989). In this context, notes produced by learners serve as “external storage” for subsequent review and study. According to a meta-analysis, reviewing notes produces overall large positive effects on performance (Henk & Stahl, 1984). Substantial evidence has demonstrated that students who reviewed notes (including notes provided to them) showed superior performance on measures of learning than students who did not review notes (Kiewra, 1985b, Kiewra et al., 1991, O'Donnell and Dansereau, 1993, Rickards and Friedman, 1978).

#### 1.1.3 Assessing quantity and content of the notes associated with successful and unsuccessful learning

More recently, research on note-taking has developed beyond experimental studies testing the relative importance of the encoding and external storage functions and has begun to delve into the quantitative and qualitative differences of notes taken by students that are associated with successful and unsuccessful learning.

##### 1.1.3.1 Note quantity and academic performance

Multiple studies have examined note-taking quantitatively, demonstrating that increased lecture note-taking (Cohn et al., 1995, Kiewra and Benton, 1988, Kiewra and Fletcher, 1984) and text note-taking (Slotte & Lonka, 1999) (e.g., measured by indicators like word count or number of important ideas in notes) are significantly positively associated with learning and test performance, whether or not students review the notes.

In addition to the extensive research that examines the quantity of notes encoded and its importance for learning, some studies on the repetition effect have investigated whether increasing the quantity of reviewing episodes can boost performance or not (Annis and Annis, 1987, Bromage and Mayer, 1986, English et al., 1934). These studies suggest that reviewing instructional material multiple times improves performance over listening to or reading instructional material during one single period. However, it is worth pointing out that the review sessions of lectures or texts in these studies are somewhat different from reviewing notes. When reaccessing and reviewing this type of instructional content, students listen to the entire lecture or reread passages. During note-reviewing periods, students reaccess and restudy their notes, which typically have lower completeness and accuracy of information, but usually contain chunks of information that they regard as important and may include notes reflecting the students’ own understanding. Meanwhile, these studies on the repetition effect mainly focus on review sessions after the study is over while reviewing notes could occur during learning to assist with real-time problem solving, especially in computer-based learning environments. Therefore, more research should be conducted on the quantity of note-reviewing, including the frequency of reviewing notes as external storage and the amount of time spent on reviewing notes, not only after the study but also during study sessions. Further, it could be useful to explore how note-takers should distribute their time between taking and reviewing notes.

Nevertheless, there is no direct measure of note-reviewing quantity as it is difficult for researchers to know whether students were reviewing notes when they accessed their notes or not. In the present study, we created a measure named note-reaccessing, representing actions where students reaccessed their notes, in many cases to retrieve information that was previously recorded in the notepad (i.e., external storage function). We studied reaccessing notes as external storage, and view this behavior as a precursor to note-reviewing since reviewing notes requires accessing them first.

In this study, we aim to address these questions by exploring the relationship between the frequency and duration of note-taking and note-reaccessing actions and student performance in an open-ended learning environment.

##### 1.1.3.2 Note content and academic performance

In addition to the quantity of notes, the content also influences academic achievement (Peverly et al., 2007). Content differences in notes may reflect different levels of cognitive processing (Craik & Lockhart, 1972), ranging from superficial verbatim copying of information to a relatively deeper level of cognitive processing that entails elaboration of instructional content (e.g., through inducing inferences, summarizing, generating hypotheses, constructing connections, self-questioning, concept mapping, etc.). Generative and elaborative note-taking (referred to as constructive by Chi, 2009) that involves deep cognitive processing in notes such as inference generation was found to predict superior performance than note-taking that involves a relatively shallower level of processing such as verbatim copying in both lecture note-taking (Armbruster, 2009) and text note-taking (Lahtinen et al., 1997, Slotte and Lonka, 1999). However, elaborative note-taking can be difficult and, as Kiewra and Fletcher (1984) have found, even undergraduate students can have difficulties in taking content elaborative notes despite being instructed to do so.

These results on the advantage of elaborative note-taking are consistent with the well-documented literature on the generation effect (Foos et al., 1994, Peper and Mayer, 1978, Peper and Mayer, 1986, Richland et al., 2005, Wittrock, 1974), which indicates that having learners generate information and meaning during study leads to increased retention and learning, compared to merely passively processing the information without generation. For example, note-taking is a generative activity when note-takers relate instructional material to their prior knowledge and generate new information by making inferences or constructing connections. Thus, note-taking that involves generative strategies is more effective and instrumental in learning than non-generative note-taking. This finding is also included in Chi’s (2009) Interactive-Constructive-Active-Passive (ICAP) framework. In ICAP, Chi posits that constructive activities are superior to active activities, based on this earlier evidence, which in turn are seen as better for learning than passive activities. Accordingly, she points out that the active process of taking notes, which is at minimum an active activity, can be expected to be better in terms of learning outcomes than being passive and not taking notes. Elaborating on presented information and generating information and ideas that go beyond the meaning of the original content in notes, which constitutes a constructive activity, is therefore hypothesized to be preferable to reproducing instructional content while taking notes, which comprises an active activity. We test this hypothesis in our present study.

### 1.2 Computer-based note-taking

Compared with the substantial literature on traditional paper-based note-taking that mostly predates the introduction of computers to science classrooms, computer-based note-taking is an emerging area of research with a growing number of studies (Bauer, 2008, Bauer and Koedinger, 2006, Crooks et al., 2007, Igo et al., 2005, Igo and Kiewra, 2007, Mueller and Oppenheimer, 2014, Robinson et al., 2006). Computer-based note-taking is different from traditional paper-based note-taking partly because typing speed on computers is typically faster than handwriting speed (Brown, 1988), probably resulting in a greater amount of information being recorded on computers. Previous research has found a positive relationship between transcription speed with lecture note-taking (Peverly et al., 2007) and text note-taking (Peverly & Sumowski, 2012). Additionally, the content and quality of notes recorded might also be different depending on how the notes are taken (Armbruster, 2009, Mueller and Oppenheimer, 2014). A few researchers investigated the effect of computers on student note-taking from lectures compared to paper-based note-taking (Bui et al., 2013, Mueller and Oppenheimer, 2014). Other researchers have studied note-taking of computer-based text content with online tools such as computer-based graphic organizers (Crooks et al., 2007, Igo et al., 2005, Igo and Kiewra, 2007, Igo et al., 2008, Katayama and Crooks, 2003, Robinson et al., 2006). Many of these studies involve exploring the effects of different designs of graphic organizers on learning and note-taking. For example, Igo et al., 2005, Igo and Kiewra, 2007, Igo et al., 2008 investigated how graphic organizers that allow different levels of copy-and-paste note-taking, which they claim link to different levels of cognitive processing, influence the learning of web-based text for different populations. Other studies (Katayama and Crooks, 2003, Robinson et al., 2006) found that the graphic organizers that provide partially complete information on instructional content and require learners to complete the remaining sections by note-taking are more effective than other graphic organizers in facilitating learning. However, note-taking in these contexts is different from our research, which focuses on plain text notes being typed in a notepad. Note-taking/reviewing of lectures or texts on computers is different from taking and reviewing notes from open-ended learning environments from multiple perspectives (discussed in detail in the following section).

#### 1.2.1 Open-ended learning environments for science inquiry

One of the important goals for K-12 science education is to help students develop the scientific knowledge and skills needed to actively and effectively engage in science inquiry (Kuhn & Pease, 2008). Over the past decade, open-ended learning environments (OELEs) have transformed traditional K-12 science classrooms by fostering learning of complex scientific topics and assessing science inquiry skills (Clarke-Midura and Dede, 2010, Land, 2000). OELEs are learner-centered, technology-based learning environments that support problem solving and inquiry by presenting learners with authentic contexts, complex and challenging learning tasks, and a set of tools and resources to explore and manipulate (Land, 2000, Segedy et al., 2015). In OELEs, learners set their own learning goals; generate, test, and modify hypotheses; utilize and manipulate tools and resources; construct solutions to problems and reflect on solutions and inquiry process (Kinnebrew et al., 2014, Land, 2000, Segedy et al., 2015). The open-endedness of OELEs is represented by the limited external directions provided in the environments, and the control and responsibility learners assume in their own problem-solving process — they pursue unique learning goals, create unique plans, and execute unique inquiry paths and learning sequences to accomplish learning goals (Hannafin, 1995, Hannafin et al., 1999).

Accumulated evidence shows that OELEs provide an authentic learning context and are effective in enhancing science inquiry skills, boosting self-regulated learning, and preparing students for future learning (Jiang et al., 2018, Jiang et al., 2015, Land, 2000). Popular OELEs that have been found to assist science learning include virtual environments (e.g., Clarke-Midura and Dede, 2010, Scalise and Clarke-Midura, in press), science microworlds (e.g., Gobert, Sao Pedro, Baker, Toto, & Montalvo, 2012), teachable agents (e.g., Leelawong & Biswas, 2008), games (e.g., Shute, Ventura, & Kim, 2013), and hypermedia (e.g., Azevedo, 2005). Despite the learning opportunities, the non-linearity and open-endedness of OELEs also impose challenges on learners in terms of extraneous cognitive load and greater requirements for self-regulated learning (Azevedo, 2005, Moos, 2009, Moos and Azevedo, 2008, Jiang et al., 2018).

#### 1.2.2 Taking and reviewing notes in open-ended learning environments

Note-taking in OELEs is different from note-taking from lectures or texts (on computers or by hand) in the following fashions. First, oral content or visual texts are delivered and notes are taken during lectures or text reading while the instructional information in OELEs is usually distributed over various representations (e.g., animations, texts, graphics, audios, videos, etc.). Second, the information students listen to and simultaneously take notes of during lectures is linear and transient. As such, lecture note-takers take notes under great time pressure. In this sense, taking notes from texts is more similar to taking notes from OELEs considering that neither texts nor the multimedia information in OELEs have the time restriction inherent to lectures. Students can select, process, and record the information at their own pace (Slotte & Lonka, 1999). However, the instructional information in OELEs is nonlinear while the texts are typically more linear. Third, learners explore open-ended learning environments actively and assume an active control of their learning and exploration. Accordingly, OELEs pose high demands on self-regulatory skills, which in turn imposes a high cognitive load on students (Moos, 2009; Jiang et al., 2018). The processing of a large volume of multimedia information from OELEs also has the potential to tax students’ limited cognitive processing capacity. Both of these processes may overload students and make note-taking in OELEs, which assumes cognitive resources, challenging. In contrast, texts or lectures entail reading or more passive listening of the linear content and less control by learners (O'Donnell & Dansereau, 1993). Thus, note-taking in OELEs poses different challenges to learners from note-taking from texts or lectures. Fourth, lecture note-takers have to divide their attention among simultaneously listening to the lecture, selecting important information and taking notes. This places limitations on the opportunity to generate new semantic information and ideas in notes during lectures (Kiewra et al., 1991). Taking notes of texts with no time limitation allows learners more time to process information and take generative notes (Lahtinen et al., 1997). Further, the non-linearity and the open-endedness of OELEs result in more flexibility and time for students to connect and coordinate representations from multiple disparate sources or generate connections between information and prior knowledge and record them in notes. Last, reviewing notes in OELEs or texts is different from note-reviewing during lectures, where review of notes mainly takes place after class when all notes have been taken. In OELEs or while reading texts, note-reviewing can happen concurrently with note-taking during science inquiry, as students have access to their notes in real-time to support their problem-solving. A summary of these differences between note-taking in different contexts is shown in Table 1.

**Table 1:** Differences and similarities between note-taking during lectures, note-taking from texts, and note-taking in open-ended learning environments (OELEs).

	Note-Taking during Lectures	Note-Taking from Texts	Note-Taking in OELEs
Mode of instructional content	Oral	Visual	Multimedia information distributed over various representations (e.g., animations, texts, graphics, audios, videos, etc.)
Linearity of instructional content	Content is delivered linearly and is transient.	Content is usually organized linearly but does not have the time restriction inherent to lectures. Students can select, process, and record the information at their own pace (Slotte & Lonka, 1999).	Content is nonlinear and students can select, process, and record the information at their own pace.
Learner control of processing	Entail more passive listening of the linear content and less control by learners (O'Donnell & Dansereau, 1993)	Learners assume a more active control of their learning than during lectures.	Learners assume an active control of their learning and exploration. The high requirements on self-regulated learning and the large volume of multimedia information impose high cognitive load that may make it challenging to allocate cognitive resources to note-taking.
Opportunities for generative note-taking	Learners take notes while simultaneously receiving the instructional content, which reduces the opportunities to take generative notes that connect instructional information with prior knowledge or with information transmitted earlier.	Learners have more time to process information and take generative notes.	Non-linearity and open-endedness provide flexibility and time to connect and coordinate representations from multiple disparate sources or generate connections between information and prior knowledge.
Timing of note-reviewing	Mainly takes place after class when all notes have been taken	Can happen either after note-taking or concurrently with note-taking to support learners’ real-time problem-solving	Happens concurrently with note-taking during inquiry to support learners’ real-time problem-solving

Results from studies on computer-based note-taking in OELEs are mixed, sometimes agreeing and sometimes contradicting the results found in the literature on traditional note-taking. For example, undergraduate learners in Trafton and Trickett’s (2001) study who used a digital notepad to take notes while solving scientific problems in an OELE for science outperformed those who did not use the notepad. Students who had used the notepad performed better even later, when it was no longer available, a result comparable to previous findings on the positive effects of note-taking in traditional settings. No relationship between the quantity/content of notes and performance was explored in this study. On the other hand, results contradicting traditional note-taking literature have been found in other OELEs. For instance, McQuiggan et al. (2008) had students take and review notes while engaging in science inquiry tasks and solving a science mystery in an OELE called Crystal Island. They did not find significantly different performance and learning gains between note-takers and non-note-takers. Malmberg, Järvenoja, and Järvelä (2010) examined the use of study tactics among twenty elementary school students who interacted with an open-ended multimedia environment for science. They concluded that taking only a few notes was associated with better learning outcomes compared to frequent use of study tactics such as note-taking. However, the sample size is this study was small.

A more recent analysis on note-taking in OELEs by Trevors et al. (2014) did not find any positive associations between the quantity and quality of notes and learning outcomes among college students. For example, they found that the frequency of note-taking actions was negatively associated with subsequent learning outcomes in a hypermedia learning environment, which contradicts the positive correlations between note quantity and performance found in previous research. However, in this study, note-reaccessing actions were not distinguished from note-taking actions. In addition, they coded notes qualitatively into content reproduction (notes where learners reproduce the instructional content through memorization or rehearsal strategies), and content elaboration (notes where learners elaborate on the underlying meaning and patterns of content). The number of content reproductive notes, which comprised the majority of the notes taken by students, was negatively associated with learning outcome. Meanwhile, no advantage of constructive and generative note-taking was found, as the number of content elaborative notes that involved a deep level of cognitive processing was not significantly associated with learning performance. The researchers argue that taking notes in OELEs is detrimental to learning and impedes performance because the cognitive overload caused by note-taking limits students’ exploration of the representations and the learning environment.

In another study on the same OELE, Bouchet et al. (2013) applied clustering analysis to classify undergraduate learners based on their use of self-regulatory processes and strategies. Results suggested that students with higher self-regulatory skills and higher prior knowledge tended to take fewer notes and spend less time taking notes than students in the other clusters. Despite their tendency to take fewer notes, these students checked their notes more often. In contrast, Sabourin et al. (2013) analyzed the differences in inquiry behaviors utilized by learners depending on their level of self-regulation within the OELE Crystal Island and obtained different results. Their results suggested that highly self-regulated students made better use of the resources and tools in the environment by taking notes of relevant information in worksheets than students with lower SRL skills. Low-SRL students used information and resources presented in the environment less effectively and did not record the information in worksheets. On the other hand, Taub et al. (2014) did not find a difference in note-taking strategies between undergraduate students with high versus low prior knowledge as they interacted with an OELE that teaches science.

With the lack of consensus in these studies, it is desirable for researchers to systematically probe into whether note-taking/reviewing is beneficial or detrimental for science performance in the context of open-ended learning environments and whether findings from classical paper-based note-taking literature can transfer to open-ended learning environments. Specifically, we extended the study by Trevors, Duffy, and Azevedo (2014) to investigate note-taking in an OELE for middle school science, and examined both the quantity of note-taking/reaccessing behavior and content of notes more comprehensively.

### 1.3 The present study

The purpose of this study is two-fold, involving analyses that are intended to answer two research questions. First, are the quantity of taking notes and the quantity of reaccessing notes associated with student success at science inquiry within an open-ended learning environment for middle school science? To answer this question, we first created measures related to the quantity of note-taking and note-reaccessing behaviors from interaction data in the open-ended learning environment and examined their relationships with student performance on science inquiry tasks. Second, is the content of notes taken in digital notepad related to student performance on complex science inquiry tasks within the open-ended learning environment? This question is addressed by coding the content of notes produced by students from multiple perspectives and examining their relationships with performance.

## 2 Virtual Performance Assessments

In this study, we explored an open-ended learning environment that was designed as part of the Virtual Performance Assessment Project (see Clarke-Midura, Code, Zap, & Dede, 2012). The environment, referred to as Virtual Performance Assessments within the project, was a 3-D immersive virtual environment that had the look and feel of a video game but was designed to assess middle school students’ science inquiry skills *in situ* (Clarke-Midura et al., 2009, Jiang et al., 2018, Scalise and Clarke-Midura, in press). Within the environment, students engage in authentic scientific inquiry activities by navigating an avatar around the open-ended learning environment, making observations, gathering data and saving data to a backpack, interacting with non-player characters (NPCs), reading kiosk informational pages for research, taking notes, and conducting virtual laboratory experiments. These actions are recorded automatically and unobtrusively on the back end in the form of process data (e.g., where they went and what they did in the open-ended learning environment) as well as product data (e.g., student notes and final claims).

**Fig. 1:**
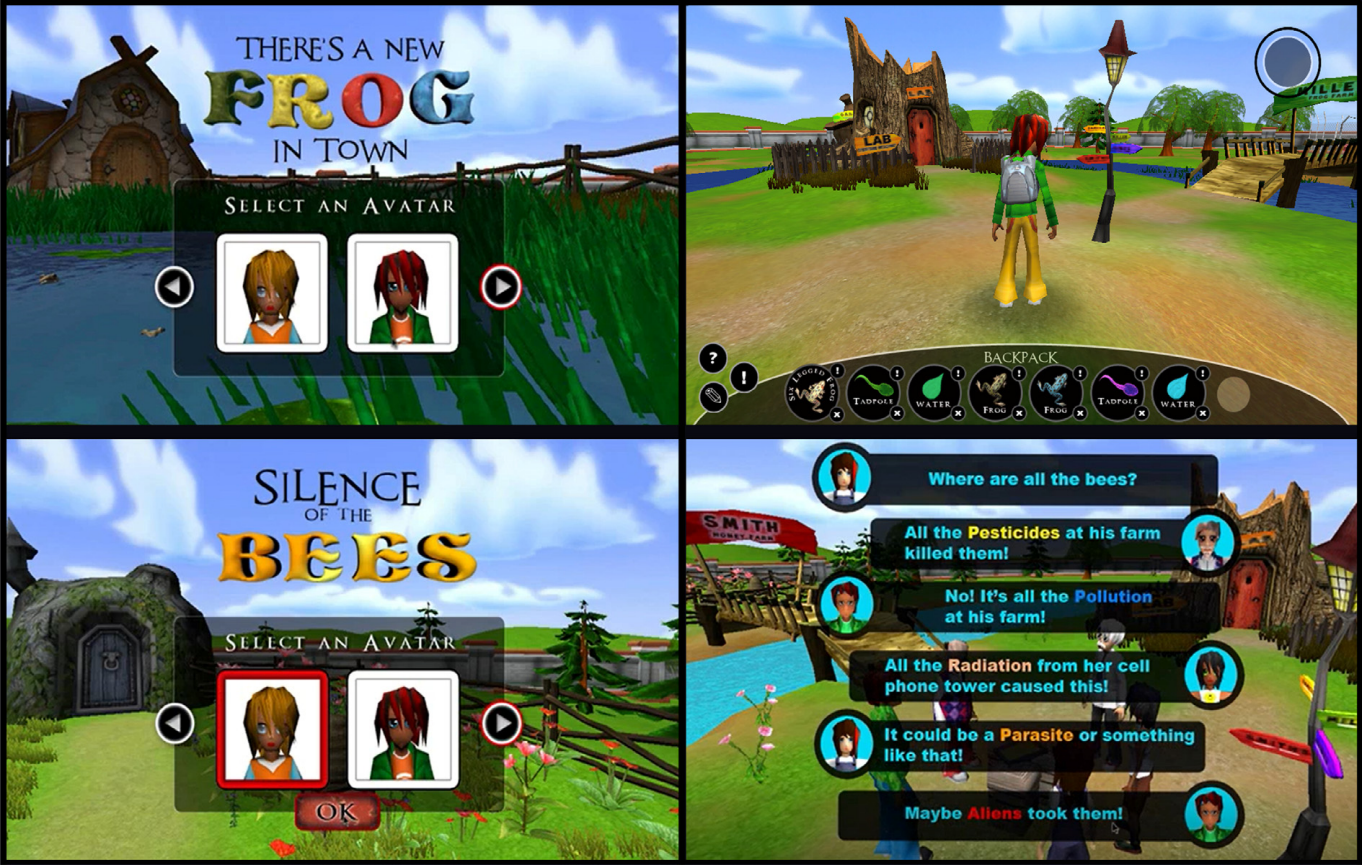
Screenshots of the frog scenario (top) and the bee scenario (bottom) of Virtual Performance Assessments.

The larger Virtual Performance Assessment Project provides students with multiple assessment scenarios. In this study, we used data from two scenarios: the frog scenario and the bee scenario (see Fig. 1). The two scenarios have similar structure and mechanics in order to allow researchers to assess performance of the same inquiry practices in different contexts. The differences between the two scenarios are the content of the problem that students are asked to solve and the surface features associated with the scenarios. In both scenarios, students visit four virtual farms to determine the cause of distress to the creature in question (frog mutation or bee deaths). In both, they are told that the possible causal factors are parasites, pesticides, pollution, radiation-induced genetic mutation, and space aliens. In each scenario, only one of these is correct.

**Fig. 2:**
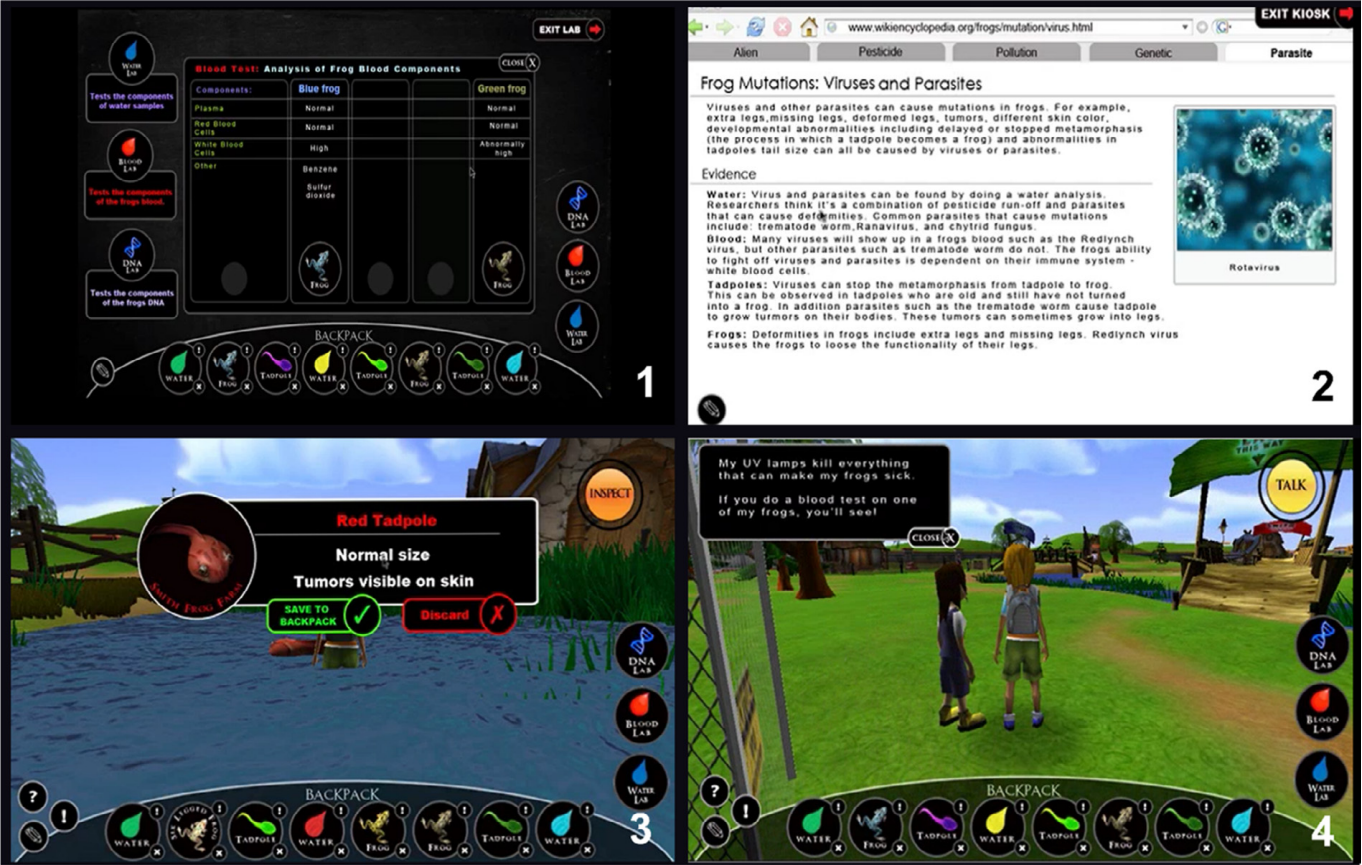
Screenshots of the different data sources in the frog scenario: (1) laboratory test results, (2) research kiosk page, (3) field observation, and (4) conversation with NPCs.

The environment contains different types of data sources. Students can talk to NPCs from the four farms who provide conflicting opinions of what is causing the problem. They can read informational pages about the five possible causal factors from a research kiosk (see Fig. 2). The information in the kiosk pages includes what types of tests and evidence can be found for each causal factor. For example, the page about parasites in the frog scenario contains information about water and blood tests and what type of results are evidence for parasites. Students can also inspect objects and samples (e.g., frogs, tadpoles, water samples in the frog scenario; bees, larvae, nectar samples in the bee scenario) from each farm and save the objects and samples they think are interesting and/or useful to a backpack. The backpack is accessible to students throughout the inquiry process. Students can add objects to or discard objects from the backpack at any time. Students can also conduct laboratory tests (see Fig. 2) such as a water analysis that includes pH levels and contaminants, a blood test that reports on components such as plasma, red blood cells, and white blood cells of the objects and samples they have saved to the backpack, and a genetic test that shows the DNA of the objects. These data provide evidence that parasites have caused the frog to grow six legs and radiation-induced genetic mutation is causing the bees to die.

**Fig. 3:**
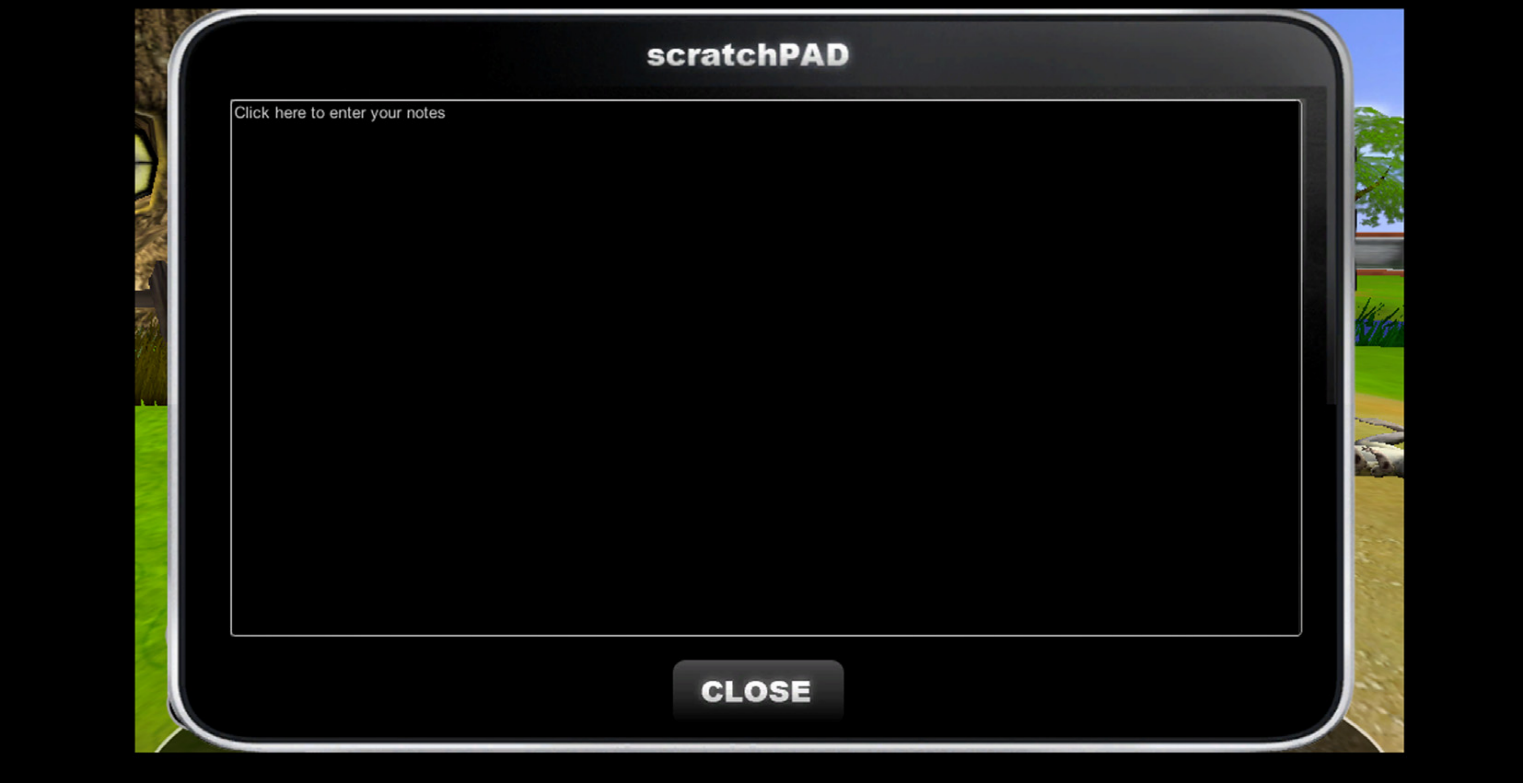
Screenshot of the digital notepad within the environment.

One of the key tools that students have as they investigate these possible causes is a digital notepad (Fig. 3). Students can access the notepad any time they want to enter information or review their notes. When taking notes in their digital notepad, students are not able to simply copy and paste information from the environment (e.g., research kiosk pages, dialogue with NPCs, laboratory test results, observation, etc.). Instead, they must hold the information they obtain in working memory and type in text in the notepad. The notepad can only contain text; there is no way for students to add pictures. The design of the digital notepad was deliberately text-based to restrict the act of copying and pasting information, forcing students to encode information. This aligns with previous research suggesting that allowing the copy-and-paste functionality limits the encoding benefits of note-taking and interferes with learning compared to tools that restrict the copy-and-paste feature (Bauer & Koedinger, 2006).

Once students think that they have collected sufficient data in each scenario, they submit a final claim on the causal factor resulting in the frog mutation or bee deaths from the list of possible hypotheses and justify their final conclusion with supporting evidence. These submissions form the primary basis of the assessment of science inquiry skills for each student.

## 3 Methods

### 3.1 Participants

For this study, 2429 middle school students (aged 12–14 years) used Virtual Performance Assessments within their 7th and 8th grade science classes at the end of the 2011–12 school year. Two other students were excluded from analysis due to lack of data on their demographics (e.g., gender). Students were drawn from 130 classrooms (39 teachers) in a diverse range of school districts in the Northeastern and Midwestern United States and Western Canada. Forty-seven percent of the students were males (*n* = 1140), and 53% of them were females (*n* = 1289).

### 3.2 Procedure

Students were randomly assigned to begin with either the frog scenario (*n* = 1232) or the bee scenario (*n* = 1197). Each student was assigned the other scenario two weeks later (bee: *n* = 824; frog: *n* = 753), subject to some attrition. Prior to each assessment, students were shown a short introductory video that provided instructions on how to use the learning environment. Following the video, students worked within each scenario until they had completed the analysis and produced a final answer for its underlying problem (e.g., why does this frog have extra legs or why are these bees dying). In sum, a total of 1985 students completed the frog scenario and 2021 students completed the bee scenario, with 1577 students completing both.

Students spent approximately half an hour in each scenario (frog: *M* = 30 min, 47 sec, *SD* = 14 min, 6 sec; bee: *M* = 26 min, 6 sec, *SD* = 12 min, 26 sec). On average, each student completed 192 actions within the frog scenario, resulting in a total of 381,331 actions. In the bee scenario, students completed an average of 196 actions, producing 396,760 actions in total. During this time, student actions, notes, and performance in the virtual assessments were automatically logged, facilitating the development of several different kinds of measures for later analyses.

### 3.3 Data sources

Three data sources were used within this study. First, student science inquiry performance within the environment was evaluated by the student’s success in designing causal explanations for why their claim was correct. Second, quantitative measures of taking notes and reaccessing notes were developed based on actions involving the environment’s digital notepad (e.g., frequency of note-taking or note-reaccessing). Third, measures of note content were developed through qualitative coding of the notes. These data sources are discussed below.

#### 3.3.1 Outcome measure—causal explanation score

As discussed above, students select one of five possible factors as the cause of the underlying problem in the particular scenario (i.e., frog mutation or dying bees). The outcome measure of science inquiry skill used in this study (hereinafter referred to as causal explanation score) evaluates both the students’ ability to pick the correct final claim and to design a causal explanation to support their claim with evidence. Most of the evidence in the frog scenario was consistent with parasites being the cause of the 6-legged frog and in the bee scenario with radiation being the cause of the death of bee population. Three of the four other incorrect claims in each scenario had at least some evidence consistent with the claim, but the evidence against them was stronger. There was no evidence in support of the aliens claim in either scenario. While the non-causal data were strong enough to show that these claims were unlikely to be the cause, students were given partial credit if they provided supporting evidence for these claims.

**Fig. 4:**
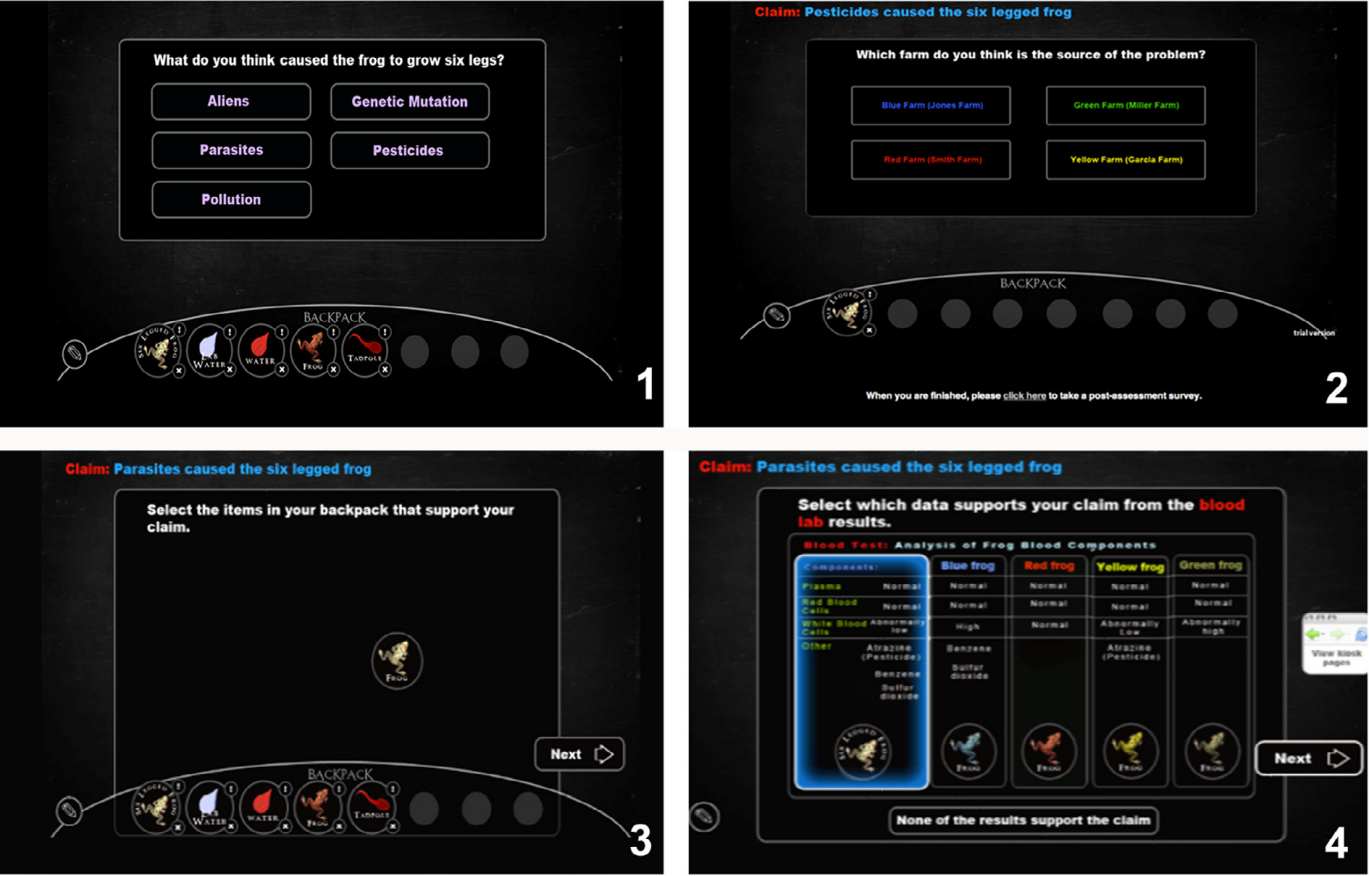
Examples of the items (final questions) that evaluate student ability in designing causal explanations in the frog scenario: (1) identification of the final claim, (2) identification of the farm that leads to the problem, (3) selection of evidence from items saved in the backpack, and (4) selection of evidence from all possible data.

The measure of students’ designing of causal explanation ability was operationalized through assigning points based on the correctness of students’ final claim and whether the evidence they provided supported the claim they made. At the end of the assessment in each scenario, students were first asked to submit a final claim on what caused the frog mutation or the death of the bee population (see Fig. 4). They were also asked to identify which farm is the source of the problem. This question helps us distinguish students who successfully teased the causal evidence from non-causal evidence and identified the problem from those who guessed the claim. Students were then asked to identify data that were supporting evidence of their final claims based on what they had collected and saved in their backpack. Last, they were presented with all possible data in the environment and were asked to identify which data supported their claim. This provided students who may not have collected all the necessary data with a chance to show that they know how to support a claim with evidence. For example, students were presented with all possible data of each type (e.g., frogs, tadpoles, blood tests, water tests, genetic tests) in separate multiple-choice questions and were asked to indicate which piece of data supported the claim they made (see Fig. 4). More details about the final questions that were used to assess science inquiry in Virtual Performance Assessments can be found in Baker, Clarke-Midura, and Ocumpaugh (2016).

In order to succeed in the environment, students must understand the causal factors and which data provides evidence for or against the particular claims in each scenario. A rubric was developed by content experts, assessment experts, and researchers in the Virtual Performance Assessment team based on how the data supported the claim. The final claim and most evidence were scored on a scale of 0–3 points based on the correctness of the claim and the strength of evidence in supporting each claim. For instance, the correct claim (i.e., parasites in the frog scenario and radiation in the bee scenario) received a full score of 3 points; the claim of aliens without any supporting evidence received a score of 0. Similarly, a piece of evidence that strongly supports the correct claim (e.g., red frog for the parasites claim) received a full score of 3 points considering that all the attributes of the red frog are consistent with the symptoms of parasites. A piece of evidence that does not support a claim (e.g., red frog for the aliens claim) received a score of 0. It is possible to receive a perfect score (3 points) when selecting evidence if the correct claim was identified, but only partial credit can be assigned for evidence for an incorrect claim due to the inherent limitations on the conclusion drawn from that evidence. For example, selecting the red frog to support the pesticides claim receives 2 points because some attributes of the red frog align with the symptoms of pesticides but there are also other attributes that are not consistent. Selecting the red frog for the pollution claim receives 1 point because the level of alignment in the attributes of the frog with the symptoms of pollution is lower. Student success in selecting their final claim and evidence to justify it was aggregated into a single composite outcome measure, by adding the points students received from all final questions and rescaling it to a measure that is scaled from 0 to 100. The data logging system kept track of all data students submitted and a back-end scoring engine automated a final score.

Therefore, even if students were unsuccessful in identifying the correct final claim, partial credit would be awarded to them for the quality and quantity of the causal evidence they identified in support of their claim from the non-causal data and results. Using this metric enabled us to better distinguish students who understood the principles of scientific inquiry (but were led astray by distractor information) from those who were completely unsuccessful at demonstrating science inquiry skills.

#### 3.3.2 Quantitative measures of note-taking/reaccessing behavior

Within the environment, students could click on the digital notepad to take or review notes. Quantitative measures representing the quantity of note-taking/reaccessing behavior were computed for each note-taker who made use of the digital notepad and were used in later analysis. Specifically, these measures were calculated based on the 1178 note-takers (59% of the entire population) in the frog scenario and the 1172 note-takers (58% of the entire population) in the bee scenario. A description of the full set of measures on notepad use behavior and their descriptive statistics can be found in Table 2. They include features that represent general notepad access, such as the number of times students opened the notepad window (i.e., notepad access frequency), the total amount of time in minutes that notepad was open (i.e., notepad time), and the percentage of total time in the environment that the student was using the notepad (i.e., percent of time on notepad). In addition, other measures were calculated by distinguishing between the encoding function (where students recorded information in notes or changed previous content) and the external storage function (where students reaccessed the notepad without adding or changing content, indicating that they likely retrieved information that was previously stored in the notepad without recording new information or editing previous notes) (Di Vesta and Gray, 1972, Kiewra, 1989). Reaccessing notes that were stored in the digital notepad can be seen as the first step of note-reviewing. While we cannot confirm that students actually read their notes when they reaccessed the notepad, it is likely that they frequently did. While other studies on note-reviewing do not address the unknown of how much focus was spent on note-reviewing, we acknowledge this in our study by using the term reaccessing, which can be seen as a precursor to reviewing. Note that note-reaccessing and note-reviewing in the present study happens concurrently with note-taking, rather than in two separate phases, and students are able to review notes to support their real-time problem-solving. As such, the note-taking and note-reaccessing behaviors in this study differ from those typically discussed in the literature, where notes are mostly taken during class and reaccessed and reviewed later. Therefore, in this case it is possible that students reaccessed the notepad as external storage without spending time to review notes. This is a limitation to our study that we will discuss later in the article.

**Table 2:** List of features related to note-taking/reaccessing quantity distilled from log data, and the descriptive statistics of the features by scenario.

Scenario		Frog	Bee
Feature	Description	*M* (*SD*)	*M* (*SD*)
Notepad access frequency	Frequency of opening the notepad window	16.13 (15.23)	15.93 (14.65)
Notepad time	Total amount of time in minutes that notepad was open	5.12 (5.18)	5.00 (4.77)
Percent of time on notepad	Total amount of time on notepad divided by total time in the environment	19% (20%)	15% (11%)
Word count in note	Number of words in note-taker’s note	59.62 (58.93)	56.97 (58.75)
Segment count in note	Number of sentence segments in note-taker’s note	8.47 (7.62)	8.31 (7.47)
Note-taking frequency	Frequency of note-taking actions	11.65 (11.22)	11.61 (11.00)
Note-reaccessing frequency	Frequency of note-reaccessing actions	4.48 (5.55)	4.32 (5.31)
Percent note-taking actions	Frequency of note-taking divided by frequency of notepad access	74% (19%)	74% (19%)
Percent note-reaccessing actions	Frequency of note-reaccessing divided by frequency of notepad access	26% (19%)	26% (19%)
Note-taking duration	Total amount of time (in minutes) spent on taking notes	4.34 (4.15)	4.30 (4.03)
Note-reaccessing duration	Total amount of time (in minutes) spent on note-reaccessing episodes	0.78 (1.71)	0.71 (1.34)
Avg note-taking duration	Average duration (in minutes) of a note-taking action	0.46 (0.43)	0.48 (0.46)
Avg note-reaccessing duration	Average duration (in minutes) of a note-reaccessing action	0.12 (0.21)	0.12 (0.22)
Note-taking to notepad time	Ratio of time spent on note-taking actions and total time on notepad	88% (14%)	88% (13%)
Note-reaccessing to notepad time	Ratio of time spent on note-reaccessing actions and total time on notepad	12% (14%)	12% (13%)

*Note*. All features were computed based on note-takers only (*n* = 1178 in the frog scenario and *n* = 1172 in the bee scenario). The mean of each feature by scenario with standard deviation given in parentheses (*M* (*SD*)) are reported.

#### 3.3.3 Measures of note content

Beyond simply studying the quantity of note-taking/reaccessing and time spent on this activity, we also analyzed the content of students’ notes, following the procedures recommended by Chi (1997). Each student’s notes were automatically parsed into sentential segments (i.e., sentence-based units) (Chi, 1997, Trevors et al., 2014), using the Stanford CoreNLP tool (Manning et al., 2014). These segments were then checked manually by the first author, who adjusted inappropriate segmentation. For example, if a student placed a period or a line break in the middle of a sentence, the sentence was manually recombined in the second-round adjustment. Similarly, comma-splices (the use of a comma to connect two independent clauses) were manually split into multiple segments. On average, students wrote 8.47 (*SD* = 7.62) segments in the frog scenario and 8.31 (*SD* = 7.47) segments in the bee scenario.

All segments were then coded by two raters using three coding schemes: (1) The *type of note* coding scheme*,* which was partially adapted from the coding scheme developed by Trevors et al. (2014), differentiated between *content reproduction* (verbatim or paraphrased content; Trevors et al., 2014), *content elaboration* (the introduction of new semantic information or ideas; Trevors et al., 2014), *metacognition* (reflection on learning process, experience, or knowledge), and *other.* (2) The *source of note content* coding scheme labeled note segments according to their origin within the system, including research *kiosks*, lab *tests*, field *observations*, and *dialogues* with NPCs. Segments which reflected a mixture of these sources were given both the label *combination* and secondary codes reflecting which sources were combined. Segments whose source could not be determined were labeled as *unknown*. (3) Finally, the *hypothesis* or *conclusion* coding scheme differentiated between segments that made a *hypothesis* about the possible causal factors for the final assessment (e.g., hypothesizing that pollution was causing the frog mutation) and segments that drew a lower-level *conclusion* from data collected (e.g., linking a farm with a bad-smelling water sample with possible pollution). Both *hypothesis* note segments and *conclusion* note segments involve *elaboration* of content presented in the environment. However, not all content elaborative notes belong to these two categories. For instance, it is possible that students tried to link two pieces of information in one segment or make an inference that was not necessarily a hypothesis or conclusion. Segments that did not belong to either of these categories were coded as *other*. Examples from the coding schemes are shown in Table 3.

**Table 3:** Coding schemes for note content. Description of each category of the measures and the relevant examples are provided.

Scheme	Category	Description	Example
Type of Note	Content Reproduction	Note segment is a verbatim copy or close paraphrase of the content presented in the environment that did not introduce new semantic information or ideas	Ethonal [sic] is a natural chemical produced by plants
	Content Elaboration	Note segment introduces new semantic information/ideas/meaning to content immediately available in the environment (e.g., making an inference, connecting information with prior knowledge, identifying underlying patterns of data, constructing internal connections, etc.)	The tadpole from Jones pond had a short tail and missing an eye, a reaction to the pesticides in the water
	Metacognitive	Note segment pertains to reflecting on and monitoring one’s own learning process, knowledge, and experience with the environment	so far the water samples that I have collected there is only one water sample that really stands out to me
	Other	Note segment does not belong to any of the other categories (i.e., Reproduction, Elaboration, Metacognitive)	all bees are starving
Source of Note	Kiosk	Note segment contains information from research kiosk pages	pesticides can cause mutations including extra limbs in frogs
	Test	Note segment contains information that could be traced to the laboratory test results	water test : pH 4.5 , atrazine
	Observation	Note segment contains information based on what students observed in the environment	yellow tadpole : smaller than normal , short tail
	Dialogue	Note segment contains information from conversation with NPCs in the environment	Another nam [sic] says that pesticides are the reason because ‘he’ sprays his fields with imidacloprid [sic]
	Combination	Note segment involves coordinating and integrating pieces of information from multiple disparate sources from the other categories (i.e., Kiosk, Test, Observation, Dialogue)	Internet Kiosk says pesticide (such as atrazine, which someone accused Garcia of using) can cause extra limbs to appear in frogs
	Unknown	Note segment contains information whose source could not be identified	i think the frog is an alien frog
Hypothesis/Conclusion	Hypothesis	Note segment proposes a possible final hypothetical claim and generates a hypothesis about the possible causal factors (e.g., pesticides, pollution, parasites, genetic mutation, aliens) leading to the mutation of the six-legged frog or the death of the local bee population	I think that the reason why the frong [sic] was abnormal and had six legs was because the water and pestisides [sic] in the water
	Conclusion	Note segment pertains to forming and drawing a conclusion from data that students collected (e.g., test results, kiosk pages, observation, dialogue, etc.)	Red bee is infected by parasites (Varroa Mites) as it has SMALL BROWN OR RED SPOTS AND STUBBY WINGS
	Other	Note segment does not belong to Hypothesis or Conclusion	frog has really low white blood

Taking into consideration the linguistic context of notes (i.e., the note segments above and below each segment), two coders (i.e., the first and the second author) independently coded all note segments from a random 10% sample of students (among those who ever took notes) in the frog scenario. Cohen’s (1960) kappa showed that substantial inter-rater agreement was achieved for the *type of note* (*κ* = .81) and the *source of note* (*κ* = .90). Results for *hypothesis*/*conclusion* (*κ* = .74) showed the need for further refinement, so definitions of each category in this scheme were further clarified in order to improve the reliability. Two rounds of coding of notes from an additional 10% of sample participants were conducted and significantly improved agreement was achieved for *hypothesis*/*conclusion* (*κ* = .90). Discrepancies in final ratings in these random samples were resolved by discussion between the raters. Once acceptable inter-rater agreement was established, the remaining note segments were then coded by the first author.

After all segments were coded, quantitative measures based on these categories were calculated for each note-taker and used in later analysis. For example, the number and the percentage of each code (e.g., *content reproduction, content elaboration,* etc.) were calculated for each note-taker, and each coding scheme, in each scenario. In addition, we computed the number and percentage of aggregated labels across coding schemes (e.g., segments coded as *content reproduction* from the research *kiosk, content elaboration* from field *observation*, etc*.*). In cases where a segment combined information from multiple disparate sources (e.g., *dialogue* and *test*), we counted this note as both a *combination* segment and as the specific categories they belonged to when calculating these measures.

### 3.4 Data analysis

Multilevel analyses were conducted to investigate the relationships between the meaningful features related to note-taking/reaccessing quantity and note content distilled from the log files (described in detail in the previous section) and student success in designing causal explanations in the environment (i.e., each student’s causal explanation score). Multilevel modeling was used because the sample consists of students nested within classes, and multiple classes that shared the same teacher. Multilevel modeling takes the hierarchy of data and the common context shared by students within classes and teachers into consideration.

Specifically, we fit three-level regression models with students in each scenario nested within classes, and classes nested within teachers. In the three-level regression models, the dependent variable is the student’s causal explanation score, and each individual feature related to note-taking/reaccessing quantity or note content serves as the single level-one predictor variable in each model. These three-level regressions were conducted for each feature to determine the relationship between the note-taking/reaccessing quantity or note content and students’ science inquiry performance after controlling for class- and teacher-level variability in each scenario. Multilevel analyses in this study were implemented using the “lme4” package (Bates, Maechler, Bolker, & Walker, 2015) and the “lmerTest” package (Kuznetsova, Brockhoff, & Christensen, 2016) in the statistical software program R.

Given the substantial number of statistical tests, we controlled for the proportion of false positives by applying Benjamini and Hochberg’s False Discovery Rate post-hoc method (FDR; Benjamini & Hochberg, 1995). This post-hoc control method avoids the substantial overconservatism found in methods such as the Bonferroni correction (cf. Perneger, 1998). Benjamini and Hochberg’s FDR method was also used in later analyses throughout this article to control for conducting multiple statistical significance analyses.

## 4 Results

In this section, we analyze the results of student use of the open-ended learning environment and its note-taking functionality, focusing on whether the quantity of note-taking/reaccessing behavior and the content of notes by middle school students are related to student success at science inquiry within the environment. Overall, 30% of students correctly selected parasites as the cause of the frog mutation in the frog scenario, while 28% of the total population correctly claimed radiation-induced genetic mutation as leading to bee deaths in the bee scenario. The mean causal explanation score for the frog scenario was higher than that for the bee scenario (*M* = 50, *SD* = 23 vs. *M* = 46, *SD* = 21).

### 4.1 Relationship between quantity of note-taking/reaccessing behavior and performance

In the frog scenario, 1,178 students (59%) spontaneously opened the notepad to take notes at least once (i.e., note-takers), and 807 students did not open the notepad to take notes at all (i.e., non-note-takers). In the bee scenario, 1172 students (58%) opened the notepad to take notes at least once and 849 students did not access the notepad at all. Three-level regressions were conducted to compare student ability in designing causal explanations between the note-takers and non-note-takers. Results revealed that the note-takers achieved a significantly higher average causal explanation score than non-note-takers (frog: *Ms* = 54 and 44, *t*(1979^a^) = 7.82, *p* < .001; bee: *Ms* = 49 and 43, *t*(2000) = 4.67, *p* < .001).

We further investigated the relationship between the quantity of note-taking/reaccessing behaviors and student causal explanation score among the note-takers only. Results with post-hoc controls are reported in Table 4.

**Table 4:** Three-level regression of student causal explanation score on each feature related to note-taking/reaccessing quantity within each scenario.

Scenario	Frog				Bee			
Feature	B	SE B	*t*	*β*	B	SE B	*t*	*β*
Notepad access frequency	.35	.04	7.87	.23^*^	.04	.04	1.00	.03
Notepad time	.73	.13	5.57	.16^*^	.21	.13	1.61	.05
Percent of time on notepad	−.63	3.28	−.19	−.01	−.32	5.82	−.05	−.002
Word count in note	.06	.01	5.05	.15^*^	.02	.01	1.70	.05
Segment count in note	.65	.09	7.46	.21^*^	.13	.08	1.54	.05
Note-taking frequency	.42	.06	6.99	.20^*^	.05	.06	.85	.03
Note-reaccessing frequency	.84	.12	6.97	.20^*^	.06	.12	.52	.02
Percent note-taking actions	−6.54	3.39	−1.93	−.05	−.88	3.19	−.28	−.01
Percent note-reaccessing actions	6.54	3.39	1.93	.05	.88	3.19	.28	.01
Note-taking duration	.81	.16	5.01	.14^*^	.20	.16	1.27	.04
Note-reaccessing duration	1.68	.39	4.28	.12^*^	.28	.47	.60	.02
Avg note-taking duration	−6.36	1.55	−4.12	−.12^*^	.28	1.32	.21	.01
Avg note-reaccessing duration	7.71	3.08	2.50	.07^*^	1.90	2.80	.68	.02
Note-taking to notepad time	−19.76	4.77	−4.14	−.12^*^	2.14	4.63	.46	.01
Note-reaccessing to notepad time	19.76	4.77	4.14	.12^*^	−2.14	4.63	−.46	−.01

*Note*. Coefficient of the predictor (B), standard error associated with the coefficient (SE B), t-statistic (*t*), and standardized coefficient (*β*) in the three-level regressions are reported. Statistically significant results after Benjamini and Hochberg’s control are marked with ^*^.

Three-level regression results indicated that measures of overall notepad use quantity (i.e., frequency of notepad access, time on notepad) among note-takers were significantly positively associated with causal explanation score in the frog scenario when controlling for the class-level and teacher-level variability (statistics are reported in Table 4). These associations suggest that the more frequently students accessed the notepad and the more time they devoted to using the notepad, the better their average performance on designing causal explanations for why their claim was correct in the frog scenario.

Results distinguishing between taking notes and reviewing notes, which comprise the two basic functions of note-taking (Di Vesta & Gray, 1972), indicated that taking notes frequently and spending more time in total on taking notes were positively associated with the causal explanation score in the frog scenario (statistics reported in Table 4). In addition, the quantity of notes (i.e., the number of words in notes, the number of sentence segments in notes) was significantly positively related to causal explanation score in the frog scenario.

Similarly, both the frequency and the duration of note-reaccessing behavior were positively associated with students’ performance on justifying the claim with supporting evidence in the frog scenario. For example, the more frequently students reaccessed notes and the more total time students spent in the notepad when reaccessing the notes they had taken, the better their average causal explanation score in the frog scenario. The percentage of time spent on reaccessing and possibly reviewing notes (relative to the time devoted to taking notes) was also positively associated with causal explanation score in the frog scenario. In other words, note-takers who spent a larger proportion of their time within the notepad reaccessing their notes without editing the notes tended to design better causal explanations in the frog scenario.

However, very different results were obtained for the bee scenario (see Table 4). In this scenario, the quantity of general notepad access and note-taking/reaccessing behaviors were not significant predictors of science inquiry performance.

### 4.2 Relationship between note content and performance

In addition to the analysis of notepad use behavior quantity, we also examined the relationship between the content of notes and student performance on the science inquiry task. For this analysis, we used the same data as discussed above, but, due to limitations in logging, seven students who deleted all of their notes before exiting the environment had to be excluded from each scenario, leaving us with 1171 note-takers in the frog scenario and 1165 note-takers in the bee scenario.

In the frog scenario, an average of 69% of a student’s note segments were verbatim copies or close paraphrases of the content presented in the environment (*M* = 6.74, *SD* = 6.99), an average of 20% of note segments were semantically elaborative notes that added new information or generated inferences (*M* = 1.36, *SD* = 2.14), and 2% of their segments were metacognitive notes. In the bee scenario, an average of 71% of student note segments were copies or paraphrases of content in the environment (*M* = 6.75, *SD* = 6.77), 21% involved content elaboration (*M* = 1.32, *SD* = 2.02), and an average of 2% contained reflective and metacognitive content. In both scenarios, a relatively large percentage of a student’s segments were based on information from research kiosk pages (39% in frog, 37% in bee), followed by notes that could be traced to students’ observation in the environment (26% in frog, 29% in bee) and notes from laboratory test results (22% in frog, 22% in bee). A relatively smaller proportion of note segments (2% in frog, 4% in bee) coordinated multiple sources of information. Specifically, most reproductive notes reproduced content from kiosk informational pages. Among the elaborative segments, students elaborated largely on observation and test results. An average of 11% of student notes from the frog scenario generated possible causal hypotheses related to the mutation of frog, and an average of 6% of student notes attempted to draw conclusions based on data. In the bee scenario, on average, 8% of student notes involved potential causal hypotheses, and 6% of student notes drew conclusions from data.

As in the previous section, we conducted three-level regressions to examine the relationships between students’ causal explanation score and the count and percentage of each category of notes, using Benjamini and Hochberg’s post-hoc control. These results are reported in Table 5. Overall, the associations between the percentage of various categories of notes and student performance were weaker than the associations between the frequency of the categories and performance. This was expected considering that the percentage of types of information will not be informative if there are relatively few notes being taken in the first place. For example, a student whose notes included ten segments, with five of them from kiosk pages (50%) could be expected to learn more than a student who only encoded one note segment and the segment was from the kiosk (100%). Therefore, we focused on the results for frequency of features.

**Table 5:** Three-level regressions of student causal explanation score on the number of different categories of segments in a student’s note within each scenario.

Scenario	Frog				Bee			
Note segment	B	SE B	*t*	*β*	B	SE B	*t*	*β*
Reproduction	.63	.10	6.59	.19^*^	.10	.09	1.10	.03
Elaboration	1.39	.30	4.55	.13^*^	.77	.31	2.54	.07^*^
Metacognition	−1.71	1.00	−1.72	−.05	−.59	1.02	−.59	−.02
Test	.87	.20	4.30	.12^*^	.66	.20	3.33	.10^*^
Kiosk	.97	.13	7.64	.22^*^	.07	.12	.56	.02
Observation	.10	.16	.65	.02	.10	.15	.67	.02
Dialogue	−.67	.51	−1.32	−.04	−.09	.42	−.21	−.01
Combination	1.61	.60	2.67	.07^*^	2.11	.59	3.61	.10^*^
Hypothesis	2.29	.60	3.79	.10^*^	1.83	.60	3.06	.09^*^
Draw Conclusion from Data	3.35	.58	5.75	.16^*^	.58	.55	1.04	.03
Reproduction of Test	.76	.25	3.01	.08^*^	.63	.24	2.62	.08^*^
Reproduction of Kiosk	.95	.13	7.16	.20^*^	.02	.13	.13	<.01
Reproduction of Observation	.15	.18	.80	.02	.03	.17	.16	<.01
Reproduction of Dialogue	−.57	.53	−1.09	−.03	−.04	.44	−.09	<.01
Reproduction of Combination	6.68	5.94	1.12	.03	2.32	1.13	2.05	.06
Elaboration on Test	2.39	.50	4.77	.13^*^	2.11	.61	3.49	.10^*^
Elaboration on Kiosk	1.77	.57	3.11	.09^*^	1.23	.58	2.12	.06
Elaboration on Observation	−.04	.52	−.08	<.01	.74	.48	1.54	.04
Elaboration on Dialogue	−5.68	3.51	−1.62	−.05	−2.32	2.95	−.79	−.02
Elaboration on Combination	1.58	.61	2.59	.07^*^	2.28	.73	3.14	.09^*^
Elaboration Test Hyp	4.18	1.30	3.22	.09^*^	4.96	1.25	3.96	.11^*^
Elaboration Kiosk Hyp	4.86	1.23	3.94	.11^*^	1.59	1.09	1.46	.04
Elaboration Observation Hyp	−1.00	1.66	−.60	−.02	3.28	1.35	2.44	.07^*^
Elaboration Dialogue Hyp	−7.57	5.41	−1.40	−.04	−2.73	6.89	−.40	−.01
Elaboration Combination Hyp	3.55	2.10	1.69	.05	3.66	1.57	2.34	.07^*^
Elaboration Test Conc	4.15	.93	4.44	.12^*^	1.52	1.20	1.27	.04
Elaboration Kiosk Conc	2.24	1.62	1.38	.04	2.02	1.17	1.72	.05
Elaboration Observation Conc	1.99	.98	2.04	.06	.44	.68	.65	.02
Elaboration Dialogue Conc	−12.51 ^a^	15.70	−.80	−.02	.13	10.36	.01	<.01
Elaboration Combination Conc	3.56	1.47	2.43	.07^*^	2.43	1.15	2.11	.06

*Note.* Significant results after post-hoc controls are marked with ^*^. Extreme values in *a* because there are few cases of Elaborative Conclusion notes based on Dialogue.

#### 4.2.1 Relationship between type of note and performance

In the frog scenario, the number of segments that involved direct reproduction of content presented in the environment was significantly positively associated with causal explanation score (statistics are reported in Table 5) such that the more students engaged in content reproduction, the more successful they were in supporting their claim with evidence. In the bee scenario, the number of content reproductive notes was not significantly associated with science inquiry performance.

Similarly, in both the frog and bee scenarios, the more note segments where students elaborated on content presented in the environment and introduced new semantic information and ideas, the better their average science inquiry performance was.

#### 4.2.2 Relationship between hypothesis/conclusion notes and performance

Generating one more hypothesis about the potential causal factors in notes was associated with a statistically significant increase of two (*SE* = 1) points in the causal explanation score in the frog scenario and an increase of two (*SE* = 1) points in score in the bee scenario. Specifically, note-takers who induced at least one hypothesis in notes performed marginally significantly better than their counterparts who did not hypothesize in their notes in the frog scenario (*M*s = 56 and 53, *t*(1140) = 2.00, *p* = .046, adjusted *α* = .035), and significantly better than the other note-takers without hypothesis notes on causal explanation score in the bee scenario (*Ms* = 53 and 47, *t*(1151) = 3.13, *p* = .002). In addition, the quantity of notes where students drew conclusions from data was also a significant predictor of student performance in the frog scenario.

#### 4.2.3 Relationship between aggregated note categories and performance

According to the results in the previous section, taking a larger amount of reproductive or elaborative notes was related to better performance in the frog scenario. In this section, we examine whether reproducing or deeply processing information from a particular resource was more strongly related to performance than other types of notes.

##### 4.2.3.1 Reproduction versus elaboration on kiosk content

In the frog scenario, the number of segments that reproduced information students read from research pages was a significant predictor of the causal explanation score (see Table 5). Elaborating on kiosk pages was significantly positively associated with the outcome measure in this scenario but with a relatively weaker association. In the bee scenario, the frequency of reproductive notes from kiosk pages was not significantly associated with causal explanation score while the number of elaborative segments on kiosk pages was marginally significantly positively associated with the score (statistics are presented in Table 5).

An omnibus likelihood ratio test and further three-level tests of pairwise comparisons with Benjamini and Hochberg’s (1995) post-hoc control method suggested the students who only copied or paraphrased content from kiosk pages in the frog scenario and those who also elaborated on kiosk pages showed significantly higher average causal explanation scores (*M* = 58, *SD* = 23; *M* = 59, *SD* = 24) than their counterparts who did not reproduce or elaborate on kiosk pages at all (*M* = 48, *SD* = 22), *t*(1166) = 5.67, *p* < .001; *t*(1161) = 5.44, *p* < .001.

On the other hand, an omnibus test indicated that the three groups (those who did not take notes on kiosk pages, those who only reproduced content from kiosk pages, and those who elaborated on content from kiosk pages) did not show significantly different science inquiry performance (*Ms* = 47, 49, and 51, $\chi^{2}$(2) = 2.64, *p* = .267) in the bee scenario.

##### 4.2.3.2 Reproduction versus elaboration on test content

On the other hand, elaborative notes that generated inferences about laboratory test results seemed to be more highly associated with causal explanation score than notes that reproduced test results. The frequency of segments where students elaborated on content from test results was positively associated with performance in both scenarios (see Table 5). The frequency of content reproductive segments based on laboratory test results was positively associated with the causal explanation score but with weaker associations.

We then compared the performance of note-takers who only reproduced test results, those who elaborated on test results, and those who neither reproduced nor elaborated on tests. Omnibus likelihood ratio tests revealed that the three groups differed significantly on their causal explanation score in both scenarios. Further pairwise tests with post-hoc control indicated that the note-takers who elaborated on test results showed a significantly higher average causal explanation score than note-takers who did not reproduce or elaborate on content from tests (frog: *Ms* = 59 and 52, *t*(1155) = 3.86, *p* < .001; bee: *Ms* = 53 and 47, *t*(1154) = 3.67, *p* < .001). On the contrary, these students who did not copy, paraphrase, or elaborate on test results did not have a significantly lower average causal explanation score than students who only took verbatim copied or paraphrased notes of the test results (frog: *M* = 52 and 54, *t*(1155) = −1.48, *p* = .139; bee: *Ms* = 47 and 49, *t*(1156) = −1.26, *p* = .208).

##### 4.2.3.3 Reproduction versus elaboration on combined content

The quantity of note segments where students copied or paraphrased content from various disparate sources was not significantly associated with performance on supporting final claim with evidence. However, the more students combined information from multiple sources and added new information and ideas to it (e.g., by generating inferences), the more successful students were at designing causal explanations.

## 5 Discussion

In this exploratory study, we applied multilevel analysis with post-hoc controls to investigate the relationship between measures related to note-taking (quantity of note-taking/reaccessing behavior, content of notes) and subsequent student success at science inquiry within an open-ended learning environment for middle school science named Virtual Performance Assessments. Overall, we found that the quantity of students’ note-taking/reaccessing behavior and specific contents of their notes tended to be positively associated with performance in the environment’s frog scenario. However, the story was more complicated for the bee scenario. In the following sections, we discuss the findings of our study as well as corresponding theoretical and empirical implications of these results for education. We conclude with a discussion of the limitations of this research and suggestions for future directions.

### 5.1 Note-taking/reaccessing quantity and performance

Results revealed that the quantity of general notepad usage was significantly positively associated with student science inquiry performance in the frog scenario, such that the more frequently students opened the notepad and the more time spent on using the notepad, the better they designed causal explanations for why their claim was correct in the frog scenario. Correspondingly, note-takers outperformed non-note-takers on science inquiry tasks, suggesting that it is advantageous to self-initiate the note-taking process and make use of the digital notepad for fostering performance on science inquiry and problem-solving. These results were also consistent with the claims by Chi (2009) that active learners who engage in taking and reviewing notes are more successful in learning than passive learners who do not take/review notes, probably because the active action of using the digital notepad for note-taking/reviewing intensifies students’ understanding of presented material and strengthens existing knowledge, which is more effective than passive processing of external information.

Thus, in the frog scenario, taking notes more frequently in the digital notepad, devoting more time to taking notes, and producing more notes (e.g., encoding more sentences or words in notes) were all associated with better performance on designing causal explanations. Taking notes more frequently and typing more notes on computers probably indicated that student attention to instructional content increased (Einstein et al., 1985), that more information was selected from the environment and transferred to text in notepad (Piolat et al., 2005), and that generative processing was involved and deeper-level mental representations of the instructional content were constructed (Bui et al., 2013, Piolat et al., 2005). These potentially help explain why taking notes in digital notepad alone was associated with better performance on science inquiry in the frog scenario. In turn, this finding also suggests that there are positive encoding benefits of note-taking and note quantity on performance, in open-ended learning environments as well as in previous research on lecture note-taking (Bretzing et al., 1987, Cohn et al., 1995) and text note-taking (Peverly et al., 2003, Rickards and Friedman, 1978). It seems that taking notes in the OELE did not limit student exploration of the environment or impede meaningful learning and performance, unlike in Trevors et al. (2014) where note-taking in an OELE was found to interfere with deep learning and was detrimental to performance.

Further, reaccessing notes more frequently and spending more time on note-reaccessing episodes, which might indicate that students were retrieving the notes they had stored in the notepad, was associated with better science inquiry performance in the frog scenario. These results indicated that the crucial role of reaccessing notes as external storage on performance found in previous literature on paper-based note-taking (Kiewra et al., 1991, O'Donnell and Dansereau, 1993) was replicated in the frog scenario. It is worth noting the different context of note-reaccessing in this work than in earlier work: within the open-ended learning environment, note-reaccessing and note-reviewing occurred in tandem with note-taking to solve a science inquiry problem in real-time, whereas in most of the previous work these two occupied separate phases, with note-taking largely being completed before note-reaccess and note-review commenced. Results also revealed that a higher proportion of time within notepad distributed to note-reaccessing episodes relative to note-taking was related to better performance on science inquiry in the frog scenario. This corresponded with previous findings that the external storage function of note-taking is relatively more important than the encoding function (Kiewra et al., 1991, Rickards and Friedman, 1978), indicating that reaccessing notes as external storage seemed to be more crucial and valuable for performance than merely recording notes. The potential contributions of the external storage function to science inquiry performance in the frog scenario as suggested by the note-reaccessing measures indicates that students should be encouraged to reaccess notes frequently in conjunction with taking notes and be provided sufficient time and opportunities to reaccess and review notes to ensure optimal inquiry performance in the frog scenario.

However, although the note-takers outperformed the non-note-takers on designing causal explanations, there was no significant relationship between the quantity of taking or reaccessing notes and science inquiry performance among the note-takers in the bee scenario. It is still unclear why the results in the frog scenario did not generalize to the bee scenario. In order to understand why differences were found in the relationships between note-taking/reaccessing and performance between the frog and the bee scenarios, it is important to understand what kinds of notes taken by students were important in these scenarios. We discuss this in the following sections.

### 5.2 Note content and performance

#### 5.2.1 Content reproduction and content elaboration

In our *type of note* coding scheme, content reproductive note segments represent what Chi (2009) refers to in her ICAP framework as active learning, contrasted with passive learning where students do not take notes when they access representations. An additional category, taking content elaborative notes, is conceptualized as constructive learning as students connect new knowledge and information with existing knowledge, generate inferences, and infer patterns and conclusions from presented content. Chi proposed in her review that constructive learning is generally superior to active learning. Overall, a majority of the notes taken by students were verbatim copies or close paraphrases of content presented in the environment, around 20% of the note segments involved introduction of new ideas and information through elaboration, and a very small proportion of notes were metacognitive. This was consistent with previous findings in classrooms that reformulated notes were rarer than verbatim/paraphrased notes (Boch and Piolat, 2005, Bretzing and Kulhavy, 1981).

According to the results, the more content reproductive note segments taken by students, the more successful they were in supporting their claim with evidence in the frog scenario. The relatively shallow level of processing that entails copying or paraphrasing content (as opposed to deeply processing the information by making inferences) was associated with science inquiry success in the frog scenario. These results contradicted previous findings that verbatim or reproductive note-taking was likely to limit exploration of the open-ended learning environment and exposure to relevant information, interfere with deep learning, and thus was negatively related with performance (Trevors et al., 2014). Although lacking deep processing, it is likely that the pure process of copying or paraphrasing content from the environment to the digital notepad without much alteration still strengthened memory for knowledge, reduced cognitive load, increased the probability of activating relevant prior knowledge, hence leading to better performance on the science inquiry tasks. In addition, it is also possible that the review of the reproduced notes ensured the fidelity of the content, and that the students with more reproductive notes produced more complete notes, which past work has shown to be related to good learning performance (Carter and Van Matre, 1975, Cohn et al., 1995). However, this positive relationship was once again not replicated in the bee scenario.

Furthermore, the more notes students took that entailed deep processing of content presented in the open-ended learning environment and the introduction of new semantic information and ideas, the better they built causal explanations in both scenarios. This was consistent with previous research that constructive learning strategies such as elaboration lead to superior learning outcomes than active and passive learning strategies (Chi, 2009). In general, generative note-taking entails increased mental effort, construction of deeper mental representations, and a higher level of engagement in problem solving than shallower processing such as verbatim copying, thereby leading to better performance (Slotte and Lonka, 1999, Trafton and Trickett, 2001).

#### 5.2.2 Aggregated note categories

Our further analysis revealed that sometimes it was not simply the depth of processing (e.g., shallow content reproduction versus deep constructive content elaboration) or the source information of note segments alone that matters, rather it might be the combination of the type of note and the source information (i.e., deep/shallow processing of information from a certain source) that was crucial for learning performance.

For example, note-takers who only reproduced content from research kiosk pages and those who both reproduced and elaborated on kiosk pages outperformed their counterparts who neither reproduced nor elaborated on kiosk pages in designing causal explanations in the frog scenario. These results indicated that recording information from the research kiosk in the frog scenario enhanced performance beyond simply reading the research pages, probably by improving students’ understanding of the domain-specific declarative knowledge presented in the kiosk, and facilitating construction of a solid knowledge base. In the frog scenario, there was no statistically significant difference in science inquiry performance between students who only had reproductive notes on kiosk and those who also elaborated on kiosk pages, suggesting that reproducing information from research pages (not necessarily making further inferences on these information within the notes) was sufficient for successful science inquiry. In other words, different from Chi’s findings (2009), our results suggested that constructive learning on kiosk pages does not necessarily lead to better outcomes than active learning. Verbatim copying or simply paraphrasing the content from research kiosk into the digital notepad appears to have helped students acquire and consolidate basic declarative knowledge, added to their knowledge base, and prepared them for constructing causal explanations in the frog scenario. However, this pattern was not replicated in the bee scenario.

By contrast, the quantity of note segments where note-takers elaborated on content about laboratory test results was more strongly associated with science inquiry performance than the quantity of note segments that only reproduced test results. Note-takers who added semantic information to test results and elaborated on the content seemed to outperform note-takers who neither reproduced test content nor elaborated on test results. There was no statistically significant difference in performance between students who only had reproductive notes on tests and those who did not reproduce or elaborate on content from test results. These results suggested that merely copying or paraphrasing the results after conducting laboratory tests might be helpful but not sufficient. Students should also learn to deeply process the lab results presented to them, link them with their prior knowledge, identify the patterns and underlying meanings, construct internal connections between results they recorded, and generate inferences and conclusions based on the test results, which will activate deep cognitive processing and stimulate constructive learning. That is, for test results, constructive learning where students elaborate on tests is related to better science inquiry performance than active learning, where notes taken by students are mainly verbatim copies or close paraphrases, as deeply processing test information allows note-takers to strengthen their understanding of the results and connect the results with the problem needed to be solved in the environment. Therefore, beyond merely reading test results, students should be encouraged to elaborate on the test results in order to promote their understanding and interpretation of the results and help them realize the connections between the test results and the problems to be solved.

Similar to notes on test results, elaborating on information collected from multiple sources in the environment was more strongly associated with performance than merely reproducing combined information. That is, simply putting information from various sources together in notes was not sufficient. Students also needed to elaborate on the internal connections between the noted information to achieve the best science inquiry performance. This finding suggested the importance of organizing and synthesizing information from disparate sources in notes and reconstructing internal connections across various categories of information for science inquiry performance in the environment.

#### 5.2.3 Hypothesis/Conclusion notes

Generating more hypotheses or drawing more conclusions in notes, which also reflects constructive learning (Chi, 2009), was positively associated with performance. This echoes McQuiggan et al.’s (2008) finding that high-performing students tended to generate hypothesis in notes within an open-ended learning environment. When teaching about note-taking strategies in OELEs, students could be taught to think more deeply about the content and construct hypotheses and conclusions in their notes to assist with science inquiry.

### 5.3 Differences between frog and bee results

The pattern of results seen in this paper was markedly different between the frog scenario and the bee scenario, two scenarios designed with the original goal of being highly similar. While there were many positive associations between measures on the quantity of note-taking/reaccessing and content of notes and science inquiry performance in the frog scenario, in the bee scenario, these features on note-taking/reaccessing quantity were not significantly associated with differences in performance. Content elaboration notes (especially from the tests and combined content), notes from tests and combined sources, and elaborative hypothesis notes (especially based on tests, observation, and combined sources) were positively associated with science inquiry performance in the bee scenario.

We postulate that the differences in results between the two scenarios are most likely caused by the differences in the design of the two learning contexts, despite similar design goals. First, we hypothesize that there are aspects in the design of the open-ended learning environment that make it more difficult for students to infer and justify the causal factors in the bee scenario than in the frog scenario, as indicated by the relatively lower average performance in the bee scenario than the frog scenario. Among the 2429 participants, 1232 students were randomly assigned to complete the frog scenario first, and 1197 students were assigned to the bee scenario as their first assessment. A three-level regression comparing the performance of these two groups in their first assessment indicated that students showed a significantly higher causal explanation score on average (*M* = 50, *SD* = 23) in the frog scenario than the performance of students in the bee scenario (*M* = 44, *SD* = 19), *t*(2390) = 5.76, *p* < .001. Meanwhile, students spent significantly more time in the frog scenario than in the bee scenario (*M* = 30 min. 56 sec., *SD* = 14 min. 24 sec. vs. *M* = 27 min. 43 sec., *SD* = 11 min. 56 sec.), *t*(2402) = 5.36, *p* < .001. That is, students tended to spend less time in conducting scientific inquiry in the bee scenario and their performance on designing causal explanations was lower in this scenario than in the frog scenario. It is possible that the difference was due to the fact that students were more familiar with the concepts and terms used in the frog scenario (e.g., water sample, blood test, pH level, etc.) compared to those in the bee scenario (e.g., nectar sample, larva test, etc.), or that the design of the evidence and counter-evidence associated with different claims in the two scenarios were different in terms of complexity. Accordingly, it is possible that more cognitive effort is required to solve the scientific problems in the bee scenario, while students did not distribute sufficient time to the inquiry and problem-solving process in this scenario. On the other hand, students engaged in a similar amount of note-taking in both scenarios, as indicated by the quantitative and content measures of note-taking. With note-taking occupying a similar amount of cognitive effort in the two scenarios, students might not have sufficient working memory space to attend to the scientific inquiry and self-regulated learning in the bee scenario, if it demanded more effort than the frog scenario. Consequently, the effects of note-taking and note-reaccessing on science inquiry performance were limited in the bee scenario as compared to the frog scenario. Second, the higher amount of time devoted to the frog scenario might suggest that students were more motivated in this scenario, considering the similar amount of information presented in the two environments. In the frog scenario, students were supposed to find which factor had caused the frog to grow six legs. In the bee scenario, they had to figure out what was causing the bees to die. It is possible that the topic and concepts related to a frog growing six legs were more concrete and interesting to middle school students than the topic and concepts involved in bee death. Motivation has been found to be related to note-taking (Moos, 2009). The different results for the two scenarios may, therefore, be related to a difference in motivation. Third, we posit that different levels of cognitive processing are required to solve problems in the two scenarios. In the bee scenario, the scientific problem is slightly more abstract and difficult, and only deep-level thinking and cognitive processing, which is more reflective of constructive learning, leads to identification of the correct final conclusion and justification of the claim with evidence. By contrast, probably because the frog scenario is relatively easier and less complex, both relatively superficial cognitive processing (e.g., through verbatim copying or closely paraphrasing information presented in the environment) and deeper-level elaboration in notes were beneficial for subsequent learning performance in this scenario. Accessing, understanding, recording, and reviewing more facts (e.g., research information), without necessarily making inferences and elaborating on them, will assist with science inquiry performance in the frog scenario. This would explain why both reproductive notes and elaborative notes were associated with science inquiry performance in the frog scenario, while mainly elaborative notes that entailed constructive learning was related to performance in the bee scenario. This difference was not intended in the original design and indicates how difficult it is to generate truly isomorphic problems in complex learning contexts such as Virtual Performance Assessments. Fourth, we speculate that the types of knowledge and information that are crucial in the two scenarios are different, leading to different results in these scenarios. It seems that the design of the environment’s bee scenario makes declarative knowledge obtained from the research kiosk less crucial for problem-solving than in the frog scenario. More specifically, information from the research kiosk is important for identification of parasites as cause of the frog mutation and justification of this claim, while the research kiosk information in the bee scenario is less essential for successful science inquiry. Therefore, note from kiosk pages was only positively related to performance in frog scenario. Considering the importance of kiosk information in the frog scenario, reaccessing and possibly reviewing the research information strengthens students’ declarative knowledge, thereby fostering performance. Correspondingly, the relative lower importance of research kiosk information in the bee scenario compared to the frog scenario might also explain partially why reaccessing the notepad, possibly to review notes was positively associated with performance in the frog scenario, but was not significantly associated with performance in the bee scenario. This hypothesis is also consistent with our result that reproducing kiosk information in notes was not significantly related to performance in the bee scenario, and the result that the frequency of reproductive notes was overall not a significant predictor of performance in this scenario.

In sum, the relationship between note-taking and science inquiry performance may be dependent on the design of open-ended learning environments. For example, the role of declarative knowledge involved in problem solving in different scenarios probably influences the relationships between learning outcomes with the frequency of reaccessing notes and the content of notes (e.g., frequency of content reproductive notes and kiosk notes). Further comparative research on the design of open-ended learning scenarios is needed to understand why the bee scenario may have been more difficult for students, and how elements in the design lead to the different results in note-taking. Similarly, the design of different OELEs (e.g., Virtual Performance Assessments and other computer-based learning environments used in previous studies) is necessary to better understand the mixed results obtained for the effects of note-taking in different learning environments.

## 6 Conclusions

In our study, we attempted to replicate previous findings on note-taking in the context of an open-ended learning environment for middle school science. However, different results were found in this study between the frog scenario and the bee scenario. In the frog scenario, the quantity of note-taking and quantity of note-reaccessing were both significantly positively associated with science inquiry performance, suggesting that the benefits of both taking notes in digital notepad (encoding function) and reaccessing notes as a precursor to note-reviewing (external storage function) on facilitating science inquiry performance within the environment. That is, the two functions of note-taking seemed to extend beyond traditional simple learning measures, to boosting performance on complex science inquiry tasks in the open-ended learning environment. These results corresponded to Chi’s claim that active note-taking is superior to passive learning, and contradicted previous research showing that taking notes in computer-based notepad and the quantity of note-taking in computers were negatively associated with performance because of the cognitive overload imposed by OELEs (e.g., Trevors et al., 2014). On the other hand, the quantity of note-taking/reaccessing behaviors was not significantly associated with performance among the note-takers in the bee scenario.

In addition, constructive learning (e.g., through making inferences in notes, combining disparate sources of information in the environment, and hypothesizing or drawing conclusions in notes) was related to better performance, in line with Chi’s ICAP framework. However, the predictions of Chi’s ICAP framework were only partially supported in the present study because the advantage for content elaborative note-taking over content reproductive note-taking was conditional on the source of notes taken. In particular, constructive elaboration appeared to be valuable for science inquiry performance when students took and reviewed notes from test results, while content elaborative notes were not necessarily superior to content reproduction when students took notes on the research kiosk information.

### 6.1 Theoretical and empirical implications

This research extends the existing note-taking literature by examining note-taking within an open-ended learning environment that assesses middle school students’ science inquiry skills in authentic classroom settings. Therefore, this study tests the robustness and generalizability of a broad list of findings from traditional research on note-taking and adds to the literature on computer-based note-taking in OELEs for science inquiry, which comprises a common learning activity nowadays.

This piece of work also shows the value of analyzing the rich log files from online environments such as Virtual Performance Assessments. In this paper, we distilled quantitative features that represent both the quantity and content of notes from log data produced by around 2000 students, enabling a comprehensive analysis of note-taking behavior in this environment. We revised and enriched the existing coding scheme developed by Trevors et al. (2014) to enable analysis of the content of notes more comprehensively than in past work in open-ended learning environments.

This study’s findings may also be of value to educational practice. Considering the importance of note-taking/reaccessing on science inquiry performance at least in some contexts, as indicated by this study and previous studies, it is critical for students, especially younger learners who lack sufficient note-taking/reviewing strategies and sophisticated self-regulatory skills (Greene et al., 2008, Pintrich and Zusho, 2002), to receive instruction and scaffolding on how to take and review notes effectively in OELEs (Bonner and Holliday, 2006, Peverly et al., 2003, Piolat et al., 2005). Nevertheless, in classrooms, most students are not provided appropriate instruction on note-taking/reviewing strategies (Bonner & Holliday, 2006). This study provides evidence on which strategies may be most effective. For example, our results imply that prompting students to reaccess notes as external storage frequently and encouraging them to spend sufficient time reviewing notes in the digital notepad might be beneficial for academic success. Alternatively, students can be encouraged by computer agents in real time to type more notes in order to promote their understanding and learning if the system detects low word count in notes. In addition, students should be instructed to evaluate the reliability of information and think critically as they take notes so that content from high-reliability sources will be prioritized and recorded – focusing on the information from the kiosk and tests rather than contradictory and possibly ill-informed NPCs. Adaptive scaffolding can also be embedded to guide students to take notes of high-importance information. For instance, cues and prompts can be provided in the environment as students read kiosk pages or test results to remind them to take and reaccess notes, while such scaffolds will not be provided as students make field observations or talk with NPCs.

On the other hand, the lack of positive associations between note-taking/reaccessing and science inquiry performance in the more difficult bee scenario indicated that taking and reaccessing notes may not be as beneficial in environments that are more cognitively demanding. In these environments, students, especially the low-performing students, should be encouraged to focus their mental effort on the science inquiry tasks and ensure that sufficient time is distributed to the inquiry process itself before encouraging them to take and use notes. Perhaps the evidence from their inquiry process could be collected for them by the environment, for later review.

Further, results from this study provide insights into where instructional designers should embed scaffolding to stimulate constructive learning and deep cognitive processing. Instead of being encouraged to take elaborative notes all the time, students should be asked to elaborate on certain data sources (e.g., connect content on lab tests with existing knowledge) in the environment. On the other hand, when note-taking is used mainly for recording declarative knowledge (such as information from the research kiosk) that can be reviewed later, elaboration is not necessary. Therefore, scaffolding on copying or paraphrasing relevant information would be sufficient in these cases to increase students’ declarative knowledge base. Results also suggested that only content elaborative notes were positively associated with science inquiry in the more difficult bee scenario while content reproductive notes were not. As such, scaffolding to stimulate elaboration could be important in the bee scenario.

Open-ended learning environments like Virtual Performance Assessments impose a high cognitive load on learners’ working memory. Previous research suggests that individual differences in working memory ability may be associated with individual differences in note-taking strategies and the effectiveness of note-taking (Bui & Myerson, 2014). Our results indicated that the effects of note-taking on science inquiry performance could be different, depending on the amount of cognitive load imposed by the learning scenario. Thus, for students with relatively low working memory capacity, scaffolding could be provided to reduce the cognitive demands on students as they learn in challenging OELEs so that the optimal amount of cognitive load is imposed. For instance, scaffolds that minimize split attention could be used to reduce extraneous cognitive load for students with low working memory capacity, potentially helping them focus on the inquiry tasks and thus maximizing the benefit of note-taking.

### 6.2 Limitations and directions for future research

One of the limitations of this study is the lack of robustness in the results. Most of the positive relationships between note-taking/reaccessing and performance found in the frog scenario were not replicated in the bee scenario, which was highly structurally similar to the frog scenario. As a result, many of our findings were only partially supported. Further investigation should be conducted with content experts and instructional designers to examine whether it was the design of the bee scenario or other elements specific to the bee scenario that caused the different results. Investigating the relationship between the difficulty level of instructional content, cognitive load, and the effectiveness of note-taking/reaccessing would also be meaningful to understand the different results. This would also elucidate the instructional design of open-ended learning environments to maximize the benefits of digital note-taking.

In addition, replication and extension of our results should be conducted to validate the generalizability of these results across platforms, domain topics, populations, and types of tasks. For example, Trevors et al. (2014) suggested in their study on note-taking in a hypermedia learning environment that the quantity of note-taking and the quantity of content reproductive notes were negatively associated with performance, while we obtained the opposite results in the frog scenario. Additionally, the present study examined the relationship between note-taking and science inquiry performance for middle school students, while most of the previous research on note-taking has focused on older populations (e.g., undergraduates and adults). Researchers have found that even undergraduate learners have difficulties in applying self-regulatory strategies such as note-taking strategies effectively and may need additional scaffolding (Kiewra and Fletcher, 1984, Moos, 2009, Peverly et al., 2003, Piolat et al., 2005). As such, it is worth asking whether the relationships between note-taking and science inquiry performance we observed among middle school students, who typically exhibit less sophisticated note-taking and self-regulated learning skills, also apply to older adults. Therefore, further analyses testing whether our results transfer to other science open-ended learning environments, for older and more proficient learners, on a variety of learning tasks, would be extremely informative and may help us understand whether younger students need different support for note-taking and note-reviewing than older students.

Another possible limitation of the study is that we have focused on note-reaccessing, which we defined as opening the notepad without adding or changing the content of notes, as an indicator of the external storage function and precursor to note-reviewing. It is worth asking how closely our findings link to the broader behavior of note-reviewing. Although reaccessing notes is the first step for note-reviewing, it does not necessarily mean that students were reviewing notes. For example, a student might rapidly open and close the notepad without spending time reading the notes. Alternate operationalizations, such as opening the notepad for a minimum amount of time, could be considered. It is also possible that a student also reviewed notes before or after entering information in the notepad while the notepad remained open. Future analysis could combine log data with eye-tracking data to provide a more valid and reliable measure of the frequency of reviewing notes that excludes actions where notes were accessed but not reviewed.

We should, of course, note that no causal inferences can be made from this correlational study. For example, in addition to the benefits of notepad usage indicated by its positive relationship with performance, it is also possible that students who accessed the notepad differed systematically and shared certain characteristics (e.g., high prior knowledge) that led them to make use of the notepad. Previous research has indicated that prior knowledge plays a crucial role in the relationship between note-taking and performance (Moos & Azevedo, 2008). However, Virtual Performance Assessments was designed to assess science inquiry skills that were otherwise difficult and complicated to measure, and it is challenging to obtain a valid measure of prior knowledge on science inquiry. Therefore, we did not collect prior knowledge on science inquiry in this study and did not control for prior knowledge while exploring the relationship between note-taking and science inquiry performance.

Furthermore, the online notepad provided to learners in this study is a plain text editor where students can enter any text. No additional features of existing popular note-taking applications such as collaborative note-taking, annotation, providing skeletal outlines for note-taking, and creating hierarchical lists, have been embedded within the notepad (cf., Bauer and Koedinger, 2006, Kauffman et al., 2011). In addition, students cannot create graphs in the notepad, which could be easily achieved in paper-based note-taking. Potential future research includes exploring the effects of enabling these features on note-taking and performance.

Research has also suggested that note-taking is influenced by individual differences. For instance, gender difference exists in traditional paper-based note-taking (Cohn et al., 1995, McQuiggan et al., 2008, Williams and Eggert, 2002). Motivation also plays an important role in why students take notes and how notes influence learning (Moos, 2009). Future research should also include examining the role of individual differences (e.g., gender, intrinsic motivation, working memory) in both the quantity of note-taking/reaccessing behavior and content of notes in open-ended learning environments.

Finally, the current study examined note-taking/reaccessing behavior within each scenario that lasted for approximately 30 min. Future studies could explore how computer-based note-taking strategies develop over time, and how they are related to delayed learning outcomes and robust learning.

## Footnotes

a Satterthwaite approximations are applied to degrees of freedom for t-tests.
